# Comparative Analysis of Three Different Impression Techniques for Impression Defects and Dimensional Accuracy Using a Digital Intraoral Scanner for Fixed Partial Dentures: An In Vivo Study

**DOI:** 10.7759/cureus.38461

**Published:** 2023-05-02

**Authors:** Neha G Khatuja, Pratibha Katiyar, Fauzia Tarannum, Kaushik Kumar Pandey, Abhishek Kumar Katiyar, Zoya Afzal, Aman Ali, Ispita Roy

**Affiliations:** 1 Prosthodontics, Career Post Graduate Institute of Dental Sciences & Hospital Lucknow, Lucknow, IND; 2 Prosthodontics, Hind Institute of Medical Science, Lucknow, IND

**Keywords:** two step without spacer, two step with spacer, single step double mix, dimensional accuracy, impression defects

## Abstract

Aim: To evaluate the impression defects and compare the dimensional accuracy of three different impression techniques (single-step, two-step without spacer, two-step with spacer) for fixed partial dentures using a digital intraoral scanner in the anterior maxillary region.

Materials and methods: Thirty subjects, above the age of 18 years with maxillary central/lateral incisor requiring fixed prostheses were selected according to the inclusion and exclusion criteria. The impressions were rated and evaluated using Heine C2.3K Binocular loupes (Heine Ltd., Dover, NH, USA). An intraoral scanner and digital vernier calipers were used to study and compare the dimensional accuracy of all three impression techniques.

Results: Statistical analysis using the chi-square test revealed that the single-step double mix technique showed the least number of defects (40%), followed by the two-step without spacer (56.7%) and then the two-step with spacer (80%) impression techniques. Using Kruskal Wallis and Mann Whitney U test for dimensional accuracy, it was found that the two-step with spacer impression technique was closer to the control group (intraoral scanner) followed by the two-step without spacer and then the single-step double mix impression techniques.

Conclusion: All three impression techniques showed the presence of impression defects, mainly voids and bubbles. The single-step double mix and two-step without spacer techniques had more favourable outcomes compared to the two-step with spacer impression technique. The two-step with spacer impression technique was dimensionally more accurate compared to the two-step without spacer and single-step double mix techniques for fixed partial dentures.

## Introduction

The most common treatment modality in replacement of a missing tooth structure or teeth is fabrication of a fixed prosthesis [1]. Precise tooth preparation and accurate fit of the prostheses on prepared teeth or implant abutments are the basic needs for restoration of mutilated or missing teeth [2]. Prosthetic dentistry plays an integral role in making impressions so as to replicate oral conditions and tooth morphology. Impression-making remains a challenging procedure due to the potential for voids and tears, which may adversely affect the precise fabrication of indirect restorations [3].

The journey towards successful restoration begins with making accurate impressions [4]. With proper material selection and manipulation, accurate impressions can be obtained for fabrication of tooth-supported restorations [5]. The impression of prepared teeth must be dimensionally accurate, stable, and compatible with gypsum products and also must reproduce the surface details of prepared teeth, all of which are prerequisites for a successful restoration [6].

The accuracy and dimensional stability of impression are in turn dependent upon impression materials and techniques. The fit of the final restoration is thus affected by the choice of impression technique selected for making an impression [7]. The impression technique determines the restoration of the finish line. Moreover, the significance of the margin in the longevity of restoration and the effect of the impression technique on marginal adaptation of restoration indicate the necessity of applying an accurate impression technique [8].

Fixed partial denture (FPD) impressions can be made with different techniques that are available which include the single-step technique (impression materials of two viscosities), double-step technique (impression made in two steps, using materials with different viscosities in each step), monophase technique (impression material of only one viscosity) and single copper band technique [3]. Inaccuracy of fit of a dental prosthesis, which may be either due to an inaccurate impression or dimensional changes of impressions, is still observed despite the availability of the best impression materials and advanced impression techniques [5].

Among the studies done on dimensional accuracy of fixed partial denture impressions, it was observed that with the improvement of impression materials, the impression technique used rather than the material itself has a greater influence on the dimensional accuracy. There are still many controversies that exist despite several studies on the accuracy of impression materials and/or impression techniques [9]. Clinical studies evaluating the clinical success of impression-making are very minimal in contrast to the wide variety of impression techniques that have been described in the literature [3].

Hence, this study was planned to compare both, impression defects and dimensional accuracy by three different impression techniques in fixed partial dentures.

## Materials and methods

The present in vivo study was conducted in the Department of Prosthodontics and Crown & Bridge, Career Post Graduate Institute of Dental Sciences and Hospital, Lucknow. The study proceeded after acquiring the necessary approval from the ethical committee of the institution (CPGIDSH/22/304). Thirty subjects, irrespective of male or female, were selected among the patients coming for dental treatment in the Department of Prosthodontics and Crown & Bridge, above the age of 18 years with clinically and radiographically healthy endodontic abutment tooth requiring fixed prosthesis in maxillary central/lateral incisor. Subjects with gingival recession, anterior malocclusion, crowding, rotation, diastema, attachment loss and clinical mobility on the abutment teeth were not included in the study. After explaining the detailed procedure of treatment, written consent was taken from each subject in his/her own language.

**Figure 1:**
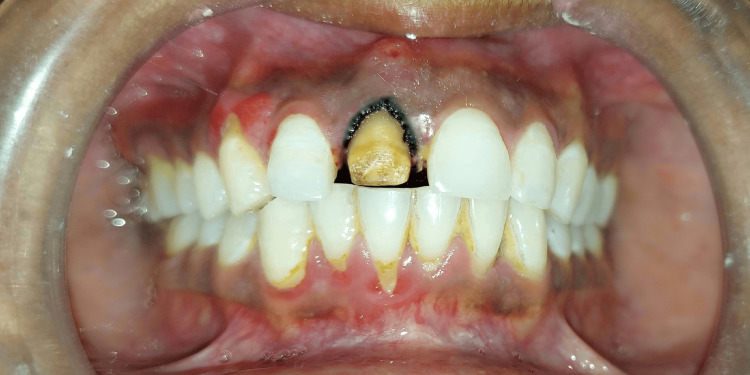
Tooth preparation and gingival retraction cord placement

Tooth preparation, followed by gingival retraction (Figure 1) using a knitted retraction cord (SureEndo; SureDent, Gyeonggi, Korea) before impression-making was done following the standard and recommended protocol [10]. A digital intraoral scanner (TRIOS; 3Shape, Copenhagen, Denmark) was used for intraoral scanning of the maxillary arch after tooth preparation of the central/lateral incisor. Using polyvinylsiloxane (PVS) putty wash impression material (Elite HD+ Putty Soft, light body, normal set; Zhermack, Badia Polesine, Italy), three impressions of each subject were made using three different impression techniques; (i) single-step double mix (ii) two-step with spacer and (iii) two-step without spacer. 

**Figure 2:**
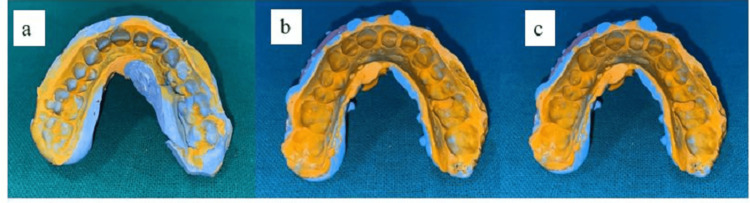
Polyvinylsiloxane (PVS) impressions made by three techniques: A) single-step double mix, B) two-step without spacer, C) two-step with spacer

For standardization of amount of impression material used and accurate placement of trays after loading of impression material, three custom trays with a spacer of 4 mm for each subject were fabricated with tissue stops (3x3 mm) over posterior sides on both sides of the arch and in the incisor area. On the side where the prepared tooth was situated, the stop was cut distal to the preparation [11]. For the single-step double mix technique, the light body PVS material was injected using an automix dispensing gun with a spiral tip around the preparation and then the custom tray was immediately seated in the patient’s mouth with putty and light body PVS material simultaneously to make the final impression and held in its place for seven minutes from the start of mixing (Figure 2A). Whereas for the other two techniques, the thick putty material was first placed in a custom tray and a preliminary impression was made. Space for the light-body "wash" material was then provided either by cutting away some of the "tray" putty material with a putty cutting knife, approximately 1-1.15 mm (two-step without spacer) or by using a thin polyethylene sheet as a spacer (two-step with spacer) between the putty and the prepared teeth (Figures 2B, 2C, respectively) [10].

**Figure 3:**
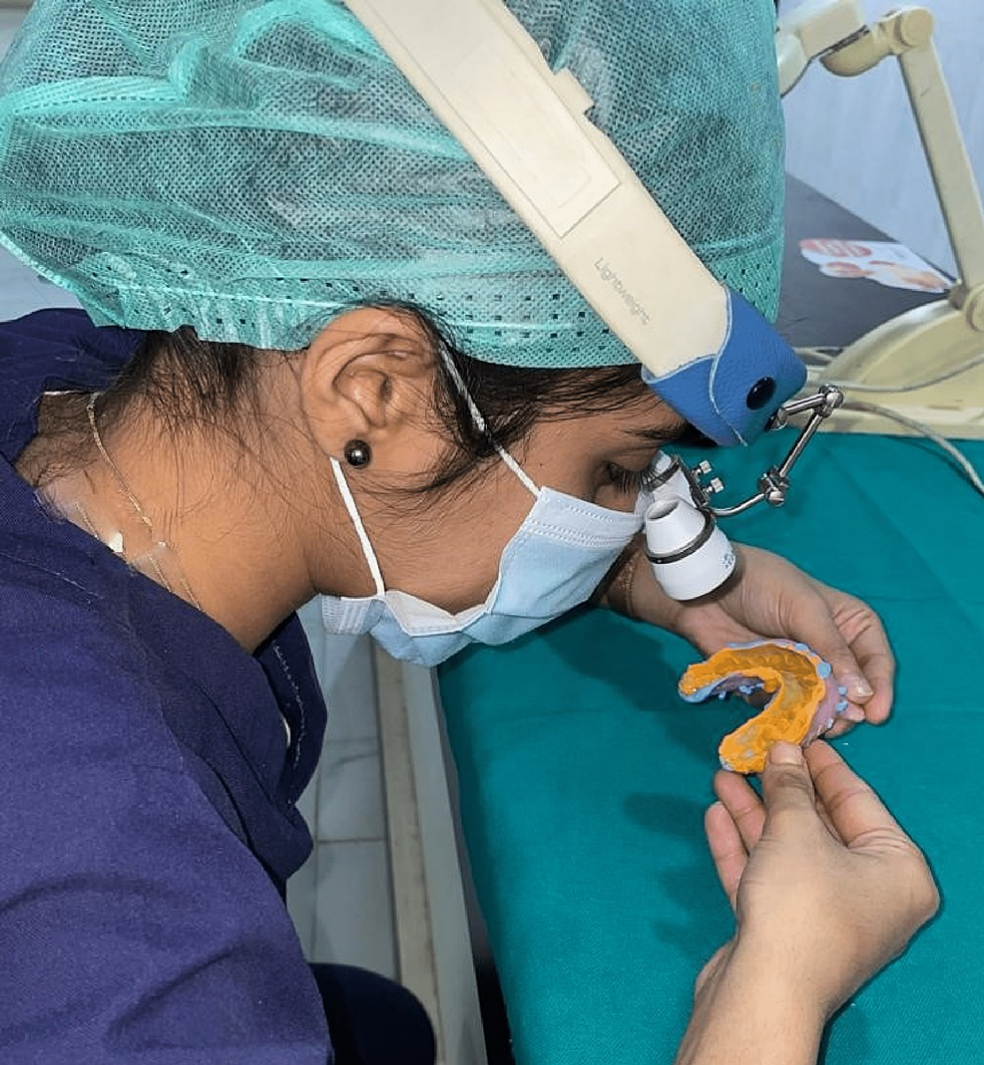
Visualization of impression defects using Heine C2.3K Binocular Loupes

All three impressions were also visually examined with the help of Heine C2.3K headband binocular loupes (Heine Ltd., Dover, NH, USA) for impression defects like voids, bubbles, tears and pull defects along with the location (Figure 3).

Impressions were rated as per the rating criteria (Table 1) where A or B was considered acceptable and C or D unacceptable [12]. Die stone (Ultrarock; Kalabhai Karson Pvt Ltd, Mumbai, India) was used to pour all the impressions and the resultant die stone model obtained was retrieved carefully avoiding damage to the cast.

**Table 1:** Rating criteria for evaluation of impression defects Permission has been granted from the original publisher [12] regarding the republication of this table.

A	No defects. Impression is reusable.
B	Small defects (tears, voids, bubbles) which do not affect finish line to prevent use of impressions. Impression is useable.
C	Good reproduction of preparation finish line. Other defects require impression to be remade.
D	Defects at preparation finish line, impression needs to be remade.
T1	Tears at the margin.
T2	Tears present in areas besides the margins.
V1	Voids present at the margin.
V2	Voids present in areas besides the margin.
B1	Bubbles present at the margin.
B2	Bubbles present in areas besides the margin.
P1	Pulls present on the lingual/palatal aspect of the impression.
P2	Pulls present in the labial/buccal aspect of the impression.
P3	Pulls present in the proximal aspect of the impression.

Measuring dimensional accuracy

**Figure 4:**
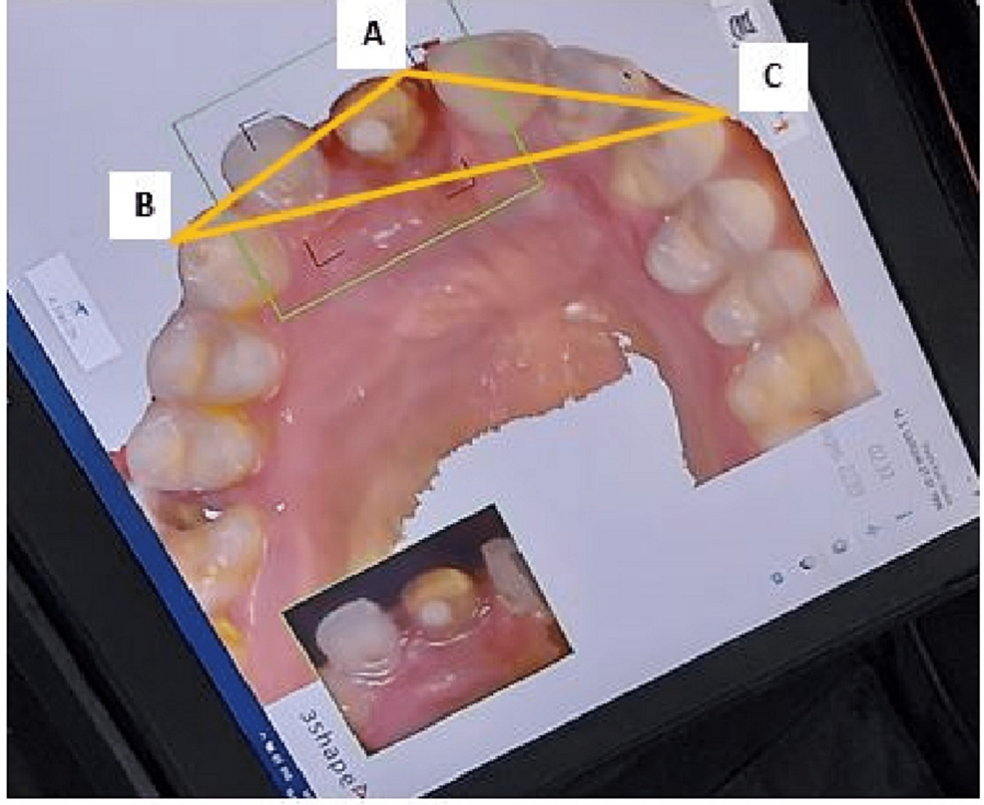
Measurement of dimensional accuracy on digital intraoral scanner AB= Distance between mesioncisal edge of prepared tooth and right bicuspid. AC= Distance between mesioincisal edge of prepared tooth and left bicuspid. BC= Distance between right and left bicuspids.

For dimensional accuracy, an intraoral scanner was used as the control group (Subgroup Ia) for obtaining maximum accuracy, and the die stone casts obtained using the three techniques (Subgroup Ib- single-step double mix technique, Ic- two-step without spacer and Id - two-step with spacer) were compared with the measurements of the control group. On the digital intraoral scan, three reference points, A, B and C, were selected where point A represented the mesioincisal edge of maxillary central/lateral incisor and points B and C represented the canine tips of the right and left cuspids, respectively. The distance between these three reference points of the intraoral scan was measured and compared with the measurements obtained by digital vernier caliper (Zhart, Jaipur, India) on the die stone models of the three impressions. The distances were measured as shown in Figure 4.

**Figure 5:**
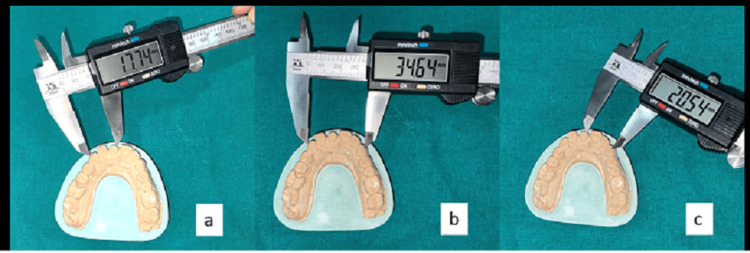
Measurement of dimensional accuracy on die stone models using digital vernier caliper: A) Distance AB, B) Distance AC, C) Distance BC

The same three reference points (AB, AC, BC) for each impression technique as the control group were considered on the die stone model. The distances on the casts were measured using a digital vernier caliper (Zhart) (Figure 5). 

## Results

On evaluation of dimensional accuracy of Subgroups Ib, Ic and Id with Subgroup Ia (Control) by using Mann Whitney U statistical test, it was found that the mean value of distances AB, AC and BC of Subgroup Id was closer to Subgroup Ia (Table 2).

**Table 2:** Evaluation of dimensional accuracy of Subgroup Id with Subgroup Ia

DISTANCE	GROUP I	Mean	Std. Deviation
AB	Subgroup Ia	18.1400	.77041
	Subgroup Id	21.0123	.72337
AC	Subgroup Ia	20.2707	.77895
	Subgroup Id	21.1883	1.80596
BC	Subgroup Ia	34.3913	.72653
	Subgroup Id	34.4550	1.70524

Comparison among the three subgroups showed significant results in the mean of distance AB (P <0.001) and insignificant results in the mean of distance AC (P = 0.006) and BC (P = 0.66) for all the three subgroups in which the mean value of Subgroup Id was closer to Subgroup Ia followed by Subgroups Ic and Ib (Table 3). 

**Table 3:** Comparison of dimensional accuracy of Subgroups Ib, Ic and Id with Subgroup Ia ***Very highly statistically significant (P<0.001), **Highly significant (P<0.01), Significant (P≤0.05), Insignificant (P>0.05)

GROUP I	DISTANCE AB		DISTANCE AC		DISTANCE BC	
	Mean	Std. Deviation	Mean	Std. Deviation	Mean	Std. Deviation
Subgroup Ia	18.1400	.77041	20.2707	.77895	34.3913	.72653
Subgroup Ib	21.9080	.76125	21.6970	1.83393	34.0133	1.65759
Subgroup Ic	21.3827	.72692	21.4110	1.80682	34.1960	1.67952
Subgroup Id	21.0123	.72337	21.1883	1.80596	34.4550	1.70524
F	153.631		4.351		.534	
p value	< .001		.006**		.66	

On evaluation of impression defects for Subgroups Ib, Ic and Id, it was found that voids and bubbles were the majority of defects seen in all the subgroups. Whereas on comparison, with the help of Chi square statistical analysis test, highly statistically significant results were found (P <0.001) in which Subgroup Ib showed the least number of defects and Subgroup Id with maximum number of defects were observed (Table 4).

**Table 4:** Comparison of defects present in Subgroup Ib with Subgroup Id ***Very highly statistically significant (P<0.05 is significant)

SINGLE STEP	WITH SPACER							Total	Pvalue
	No defect	Bubbles at margin	Bubbles besides margin	Void at margin	Void beside margin	Pull lingual	Pull labial		
No defects	6(20%)	4(13.3%)	6(20.0%)	2(6.7%)	0	0	0	18(60.0%)	<0.001***
Bubbles at margin	0	0	0	4(13.3%)	0	0	0	4(13.3%)	
Bubbles besides margin	0	0	0	0	2(6.7%)	1(3.3%)	0	3(10.0%)	
Voids at margin	0	0	0	0	0	3(10.0%)	2(6.7%)	5(16.7%)	
Total	6(20%)	4(13.3%)	6(20.0%)	6(20.0%)	2(16.7%)	4(13.3%)	2(6.7%)	30(100.0%)	

## Discussion

The fabrication of a successful prosthesis for fixed partial dentures begins with the precise replication of dental and dentoalveolar structures during the impression-making procedure. Apart from the selection of appropriate impression material, certain other factors like tray selection, tray adhesive, and impression technique also play a crucial role in reproducing accurate and dimensionally stable impressions [7].

Among the different types of elastomeric impression materials that are available, addition silicones, commonly known as polyvinylsiloxane, are widely being used in prosthetic dentistry. PVS qualities like having excellent mechanical and physical properties, excellent dimensional stability, ease of manipulation, best elastic recovery with no by-product on polymerization, facilitating pouring of impression at the convenience of the operator and allowing to make a second pour when required have made its use extremely popular in the recent practice. The advantage of the available auto mix system in these materials over hand mixing leads to less number of defects like voids, bubbles, and pull defects that might occur during the process of impression making thereby affecting the dimensional accuracy [4].

There have been several discussions among authors regarding the most critical factor affecting dimensional accuracy. According to Hung et al. [13], the choice of impression material played an important role in reproducing dimensionally accurate impressions, which was in contrast to studies conducted by Craig which stated that the impression technique that was selected was of prime importance [14].

In the current study, only maxillary central/lateral incisors were selected for all subjects as these teeth are less prone to wear and tear with advanced age and can be easily scanned intraorally with intraoral scanner as compared to the remaining teeth [1]. Digital Intraoral scanner was selected as the control group as it previsualizes the area of interest in three dimensions, reduces working time, provides accurate digital casts and decreases risk of distortion associated with the use of impression materials. The advantages of using digital vernier calipers for measurement on die stone models were that they have high reliability and accuracy, could be zeroed at any point and measurements could be easily converted if needed. Heine C2.3 K Binocular magnifying loupes were preferred for evaluation of impression defects since they showed crisp, clear images, there was flexibility for precise adjustment, and they are super lightweight and comfortable.

The results found in this study were in agreement with studies conducted by Caputi et al. [9], Nissan et al. [15] and Kumari and Nandeeshwar [16]. These findings may be attributed to the variation in the technique of impression-making. The single-step double mix technique is simple to manipulate and has reasonable economy but the major problem is the lack of control of wash bulk which leads to inaccurate reproduction of surface details including the critical areas, such as finish lines which may be enclosed in putty since the light body material from the prepared tooth is displaced by the putty thereby affecting the dimensional accuracy. Another difficulty was that once the light body material is injected on the prepared tooth prior to placement of the tray with putty and light body both, it is essential to seat the tray with the putty material in a proper position. But there are chances of removal of light body material from the prepared tooth due to the movement of the patient’s tongue or in case of elevated floor of the mouth. This may lead to inaccurate surface details reproduction of the prepared tooth [9].

On the other hand, in the two-step impression technique with or without spacer, the finish lines of the prepared teeth are recorded with the light body material resulting in better detail reproduction. The possible reason mainly lies in the ability to control the wash bulk of the impression thereby resulting in accurate casts which is not possible in the case of the single-step double mix technique. Also, after the putty has polymerized, further contraction of the light body impression material leads to only minimal dimensional changes [9].

The selection of an appropriate impression tray is one of the critical variables in the choice of impression techniques to be used [17]. In this study, acrylic resin custom trays were used instead of stock trays. This was in accordance with a study conducted by Shivakumar et al. [4]. In the putty wash impression technique, when a stock tray is used, the occlusal portion of the impression is at a variable distance and since the occlusal surface of the abutment is at a greater distance from the tray adhesive, there is greater amount of contraction of the occlusal portion of the mould space towards the tray as compared to a custom tray. This larger contraction in a stock tray would then result in increased occlusogingival height thus affecting the fit of the final prosthesis [2]. Close-fitting custom trays, on the other hand, minimize the potential cast distortion and increase the accuracy of impressions by exerting higher pressure on the impression material to record the details of the preparation. Also, use of custom trays incorporates uniform thickness of impression material with minimal wastage of material and is more convenient for patients [17].

Uniformity of the wash space also plays a crucial role in determining the dimensional accuracy of an impression [7]. In the present study, in two-step impression techniques, wash space was created by scrapping the concerned area of the prepared tooth after the initial putty impression with the help of putty cutting knife whereas another technique involved the use of polyethylene sheet as a spacer at the time of first stage putty impression. Creating space for wash material by either of the two techniques may cause distortion of the putty material during the final impression since there are chances of light body material being compressed while seating the tray. Also, during the final impression, the tray might shift from its original position thus affecting the accuracy of impression [17]. Thus, the use of polyethylene sheet as a spacer has an advantage over scrapping the impression since it provides uniform thickness of the wash material resulting in accurate impressions. Studies conducted by Gautam et al. [7] and Nouri et al. [17] supported this.

The findings in our study showed that the two-step impression technique with spacer was more dimensionally accurate than the two-step impression technique without spacer and the single-step double mix technique. This was in contraindication to studies conducted by Luthardt et al. [18] and Pande et al. [19] in which it was found that the single-step double mix technique was dimensionally more accurate when compared to the two-step technique. The possible reason for this finding may be attributed to the use of individualized metal stock trays and double retraction cord technique thus leading to more complete reproduction of the finish line of the prepared tooth. Also, obtaining slightly larger dies in the single-step technique may be advantageous as it may compensate for the contraction developed during metal casting [18].

Studies conducted by Hung et al. [13], Idris et al. [20] and Vitti et al. [21] concluded that both the single-step double mix and two-step techniques were dimensionally accurate which was again contradictory to the results found in our study. The reason for this finding may be due to insufficient magnitude, though depicting statistically differences and also neither of them resulting in dies that deviated sufficiently from the master model to cause clinically important difficulties in the fit of the castings. Another study by Caputi et al. [9] was in contradiction to our study. In this study it was concluded that the two-step injection technique was dimensionally more accurate on comparison with monophase, single-step double mix and two-step techniques with and without spacer. The possibility of this finding was that two-step with or without spacer involved the creation of an occlusal stop on adjacent teeth, as some light body material may spread along the occlusal surfaces during reseating of the putty. Also, the light body material may be displaced during this second step thus generating distortions that may lead to reduced dimensional accuracy of the impression. Another reason could be that the preliminary putty impression could be displaced by the wash material during the seating, and cause resiliency of the putty material on removal of the impression resulting in a tendency towards larger inter-abutment distances. Thus, the advantage of the two-step injection technique is that, during the first seating of the putty and wash material, there is displacement of soft tissues, such as the tongue whereas, in the second step, all of the finer details are recorded by the extra light body material without being compressed [9].

A study by Idris et al. for evaluation of impression defects revealed that bubbles are the most common defects that are seen in the single-step impression technique which can be reduced by using the two-step technique for impression making [20]. The findings in our study contraindicated this but were in accordance with studies conducted by Shrestha et al. [12] and Qadiri et al. [3]. The reasons for more voids in the two-step impression technique are that, since less amount of light body PVS material is used in the single step, so there may be less chances of voids but in the two-step technique, a large amount of light body PVS material is required that may lead to more air incorporation and may lead to increased number of voids [3]. Another reason may be attributed to the operator’s mixing technique, which could be prevented by using auto-mixing techniques. By using auto-mixing cartridges there is a tendency for fewer bubbles to be seen in impression than hand spatulation. Impression defects like tears were generally not observed in the impression because of the good tear resistance of the impression material used [3].

Further clinical studies using different impression materials, impression techniques, single and multiple abutment teeth, evaluators should be considered with a larger sample size for the complete clinical assessment of impression defects seen in PVS impression materials. Many in vitro studies have been done to determine the dimensionally accurate impression technique but there are hardly any in vivo studies that can prove the same. Due to the very limited literature available, there is a need for further in vivo studies to be conducted in order to support the findings as seen in the present study. Also, more research needs to be done on the advancements in impression techniques that can be used for impressions in fixed partial dentures.

Limitations of the study

The potential limitations of this study could be that the subjects were confined to a limited area of the dentition, the dimensional accuracy and impression defects were noted for only one prepared tooth, mandibular arch was not taken into account, only three impression techniques were taken into consideration in this study and only one brand of addition silicone impression material was considered.

## Conclusions

Within the limitations of this study, the following conclusions were drawn: On evaluation of dimensional accuracy of single-step double mix, two-step without spacer and two-step with spacer, the mean value of two-step with spacer was found to be closer to digital intraoral scanner (Control). Two-step impression technique with spacer was dimensionally more accurate when compared to two-step technique without spacer and single-step double mix technique. Voids and bubbles were the most commonly seen impression defects in all three impression techniques. Single-step double mix technique showed the least number of impression defects followed by two-step without spacer and two-step with spacer.

## Appendices

Permission has been taken from the original publisher regarding republication of Table 1.
